# The Perspectives of Mesenchymal Stem Cell Therapy in the Treatment of Multiple Sclerosis

**Published:** 2015

**Authors:** Nima Naderi


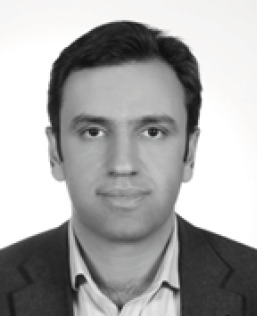


Currently approved therapies for multiple sclerosis (MS) mostly directed towards palliating inflammation, in particular in those patients with the relapsing–remitting form of MS. However, in a significant number of patients, the disease might be worsen during time. Most importantly, at present there is no therapy available to repair the irreversible neuronal damage, which leads to a progressive disability in most patients. Therefore, there is still a need for an innovative treatment that is safe and able to control the inflammatory activity leading to neurodegeneration and, more importantly, to promote repair of the damaged neurons. In such situation, stem cell therapy could be considered as an approach that worth to try due to its immunomodulatory, neurotrophic, and neuroprotective effects. Different types of stem cells have been considered for MS treatment, including embryonic stem cells, neural precursor cells, induced pluripotent stem cells, hematopoietic stem cells, and mesenchymal stem cells (MSCs). Amongst these stem cells, treatment with MSCs has been shown to be most consistent in animal models of MS. MSCs are a type of progenitor mesodermal cells that could be isolated from almost every connective tissue, but those derived from the bone marrow are the most characterized for their physiological and potentially therapeutic features. They have specific properties that allow them to escape immune rejection and have been successfully used allogeneically in human disorders. MSCs are able to modulate several functions of T and B cells, dendritic cells, and cells of the innate immune system (such as natural killer cells and neutrophils), which have a role in pathogenesis of autoimmune diseases. They also can release antiapoptotic and neurotrophic molecules that provide a neuroprotective effect in neurodegeneration condition. Specifically, MSCs can modulate the intensity of an immune response by inhibiting antigen-specific T cell proliferation and promoting the generation of regulatory T cells, which would be of great value in treatment of MS but has the adverse effect of excessive inhibition of T cell responses that would give rise to vulnerability to infectious diseases. MSCs could also exert a potent neuroprotective effect through different mechanisms, such as secretion of brain-derived neurotrophic factor and nerve growth factor, and also can prevent neurons from apoptosis. Moreover, MSCs have the ability of secreting substances that can protect neurons from excitotoxicity due to NMDA-induced of calcium influx into neurons. The neuroprotective effect of MSC may occur through an increase in the expression and release of neuroprotective molecules by microglia. Furthermore, interaction between MSCs and neural progenitor cells would give rise to more oligodendrocytes and less astrocytes (in part through suppression of astrogliosis). By reviewing the *in-vivo* studies regarding the effects of MSCs on murine MS models, it could be suggested that the therapeutic plasticity of MSCs is due to their ability to secrete paracrine substances rather than in a direct contact mechanism with damaged neurons. 

Based on the results of preclinical studies, few small phase I clinical studies have been launched since 2007, and recently an international multicenter phase II clinical trial was initiated in order to further define the safety and the efficacy of MSC therapy on larger number of patients. Results of the preliminary trials in MS suggest that MSC treatment is well tolerated and generally safe, with few less important adverse events in intrathecal injection. However, long-term safety issues of MSC are still not determined. In addition, the concern that MSC might induce tumor cell growth has not yet been addressed. In assessment the efficacy of MSC treatment, an improvement in visual evoked response latency, was reported. Overall, the results suggest that MSC are likely to find a place among the therapies for MS patients who are refractory to first-line treatments. However, larger and long-term controlled clinical studies are needed to clearly assess safety along with efficacy.

*Nima Naderi is currently working as Associate Professor of Department of Pharmacology and Toxicology, School of Pharmacy, Shahid Beheshti University of Medical Science, Tehran, Iran. He could be reached at the following e-mail address: naderi@sbmu.ac.ir*

